# Proxy measures for the assessment of psychotic and affective symptoms in studies using electronic health records

**DOI:** 10.1192/bjo.2023.623

**Published:** 2024-01-05

**Authors:** Álvaro López-Díaz, Fernanda Jazmín Palermo-Zeballos, Luis Gutierrez-Rojas, Luis Alameda, Francisco Gotor-Sánchez-Luengo, Nathalia Garrido-Torres, Johann Métrailler, Livia Alerci, Vincent Bonnarel, Pablo Cano-Domínguez, Elma Avanesi-Molina, Miguel Soto-Ontoso, Rocio Torrecilla-Olavarrieta, Leticia Irene Muñoz-Manchado, Pedro Torres-Hernández, Fermín González-Higueras, Juan Luis Prados-Ojeda, Mario Herrera-Cortés, José Miguel Meca-García, Rafael Manuel Gordillo-Urbano, Cristina Sánchez-Robles, Tomás Delgado-Durán, María Felipa Soriano-Peña, Philippe Golay, Philippe Conus, Benedicto Crespo-Facorro, Miguel Ruiz-Veguilla

**Affiliations:** Mental Health Clinical Management Unit, Virgen Macarena University Hospital, Seville, Spain; Translational Psychiatry Research Group (PsyNal), Seville Biomedical Research Centre (IBiS), Spain; Spanish Network for Research in Mental Health, Carlos III Institute (CIBERSAM, ISCIII), Seville, Spain; Department of Psychiatry, School of Medicine, University of Seville, Spain; and First-Episode Psychosis Research Network of Andalusia (Red PEPSur), Spain; Mental Health Clinical Management Unit, Virgen Macarena University Hospital, Seville, Spain; and First-Episode Psychosis Research Network of Andalusia (Red PEPSur), Spain; First-Episode Psychosis Research Network of Andalusia (Red PEPSur), Spain; Mental Health Clinical Management Unit, San Cecilio University Hospital, Granada, Spain; Psychiatry and Neurosciences Research Group (CTS-549), Institute of Neurosciences, University of Granada, Spain; and Department of Psychiatry, University of Granada, Spain; Service of General Psychiatry, Treatment and Early Intervention in Psychosis Program (TIPP), Lausanne University Hospital and University of Lausanne, Switzerland; and Department of Psychosis Studies, Institute of Psychiatry, Psychology and Neuroscience, King's College London, UK; Department of Psychiatry, School of Medicine, University of Seville, Spain; First-Episode Psychosis Research Network of Andalusia (Red PEPSur), Spain; and Mental Health Clinical Management Unit, Virgen del Rocío University Hospital, Seville, Spain; Translational Psychiatry Research Group (PsyNal), Seville Biomedical Research Centre (IBiS), Spain; Spanish Network for Research in Mental Health, Carlos III Institute (CIBERSAM, ISCIII), Seville, Spain; First-Episode Psychosis Research Network of Andalusia (Red PEPSur), Spain; and Mental Health Clinical Management Unit, Virgen del Rocío University Hospital, Seville, Spain; Service of General Psychiatry, Treatment and Early Intervention in Psychosis Program (TIPP), Lausanne University Hospital and University of Lausanne, Switzerland; First-Episode Psychosis Research Network of Andalusia (Red PEPSur), Spain; and Mental Health Clinical Management Unit, Virgen de la Victoria University Hospital, Málaga, Spain; First-Episode Psychosis Research Network of Andalusia (Red PEPSur), Spain; and Mental Health Clinical Management Unit, Torrecárdenas University Hospital, Almería, Spain; First-Episode Psychosis Research Network of Andalusia (Red PEPSur), Spain; and Mental Health Clinical Management Unit, Jerez University Hospital, Cádiz, Spain; First-Episode Psychosis Research Network of Andalusia (Red PEPSur), Spain; and Mental Health Clinical Management Unit, Jaén University Hospital, Spain; First-Episode Psychosis Research Network of Andalusia (Red PEPSur), Spain; and Mental Health Clinical Management Unit, Reina Sofía University Hospital, Córdoba, Spain; First-Episode Psychosis Research Network of Andalusia (Red PEPSur), Spain; and Mental Health Clinical Management Unit, Poniente University Hospital, Almería, Spain; First-Episode Psychosis Research Network of Andalusia (Red PEPSur), Spain; and Mental Health Clinical Management Unit, Infanta Margarita Hospital, Córdoba, Spain; First-Episode Psychosis Research Network of Andalusia (Red PEPSur), Spain; and Mental Health Clinical Management Unit, Juan Ramón Jiménez Hospital, Huelva, Spain; First-Episode Psychosis Research Network of Andalusia (Red PEPSur), Spain; and Mental Health Clinical Management Unit, San Agustín University Hospital, Linares, Spain; Translational Psychiatry Research Group (PsyNal), Seville Biomedical Research Centre (IBiS), Spain; Spanish Network for Research in Mental Health, Carlos III Institute (CIBERSAM, ISCIII), Seville, Spain; Department of Psychiatry, School of Medicine, University of Seville, Spain; First-Episode Psychosis Research Network of Andalusia (Red PEPSur), Spain; and Mental Health Clinical Management Unit, Virgen del Rocío University Hospital, Seville, Spain

**Keywords:** Schizophrenia, psychosis, affective disorders, electronic health records, clinical assessment tool

## Abstract

**Background:**

There is a lack of standardised psychometric data in electronic health record (EHR)-based research. Proxy measures of symptom severity based on patients' clinical records may be useful surrogates in mental health EHR research.

**Aims:**

This study aimed to validate proxy tools for the short versions of the Positive and Negative Syndrome Scale (PANSS-6), Young Mania Rating Scale (YMRS-6) and Montgomery–Åsberg Depression Rating Scale (MADRS-6).

**Method:**

A cross-sectional, multicentre study was conducted in a sample of 116 patients with first-episode psychosis from 12 public hospitals in Spain. Concordance between PANSS-6, YMRS-6 and MADRS-6 scores and their respective proxies was evaluated based on information from EHR clinical notes, using a variety of statistical procedures, including multivariate tests to adjust for potential confounders. Bootstrapping techniques were used for internal validation, and an independent cohort from the Treatment and Early Intervention in Psychosis Program (TIPP-Lausanne, Switzerland) for external validation.

**Results:**

The proxy versions correlated strongly with their respective standardised scales (partial correlations ranged from 0.75 to 0.84) and had good accuracy and discriminatory power in distinguishing between patients in and not in remission (percentage of patients correctly classified ranged from 83.9 to 91.4% and bootstrapped optimism-corrected area under the receiver operating characteristic curve ranged from 0.76 to 0.89), with high interrater reliability (intraclass correlation coefficient of 0.81). The findings remained robust in the external validation data-set.

**Conclusions:**

The proxy instruments proposed for assessing psychotic and affective symptoms by reviewing EHR provide a feasible and reliable alternative to traditional structured psychometric procedures, and a promising methodology for real-world practice settings.

Periodic monitoring of psychotic and affective symptoms in the early stages of psychosis is essential for improving mental health outcomes in individuals with psychotic disorders.^1,2^ Regular assessment of those symptoms is also crucial for assessing the patient's clinical course over time, examining the efficacy of therapeutic interventions and identifying early signs of relapse.^3^ There are numerous assessment instruments for the severity of psychotic and affective symptoms, including self-report, structured and semi-structured clinician-rated scales.^4^ Among the clinician-rated scales for measuring psychotic symptoms in patients with schizophrenia and other psychotic disorders, the Positive and Negative Syndrome Scale (PANSS), Brief Psychiatric Rating Scale (BPRS) and Clinical Global Impression–Schizophrenia scale (CGI-SCH) are the most widely applied.^5–7^ The Young Mania Rating Scale (YMRS), Montgomery–Åsberg Depression Rating Scale (MADRS) and Hamilton Rating Scale for Depression are among the most widely used instruments for assessing the severity of manic and depressive symptoms in patients with bipolar disorder and depressive disorders.^8–10^ Outside of the research framework, and with the exception of the CGI-SCH (which is a simple, brief instrument for evaluating severity and treatment response, but does not capture the full spectrum of psychotic features), all of these scales require significant administration and assessment time, as well as technical training for their reliable application. This usually makes them difficult to use in time-constrained settings such as clinical practice in community mental health services,^11^ and is the main reason why these psychometric instruments are seldom used for monitoring symptom severity and treatment response in large-scale, real-world research, such as electronic health record (EHR)-based studies. Furthermore, there is growing concern about selection bias in psychosis research, as findings are usually based on a subset of patients who are willing to participate in research studies.^12^

A proxy measure is a tool used when it is not possible to directly assess a construct or phenomenon of interest.^13^ Proxy measures rely on the assumption that certain variables may be a good indicator of others that cannot be or are difficult to measure directly by standardised methods.^13^ Proxy tools have great potential in health science research, since they can provide an effective and efficient alternative for the measurement of outcomes or constructs that cannot be easily collected in daily practice. Several types of proxy indicators are used in health sciences. One is psychometric assessment by close relatives or key informants (third-party assessments) when the patient cannot be directly assessed by the clinician, or when the patient is unable to report their own symptoms. Parent proxy-report versions of the MADRS and the YMRS are examples.^14,15^ Another type of proxy measure uses information from a surrogate scale replacing a more complex or time-consuming assessment tool. Some examples can be seen in the use of the National Adult Reading Test as a proxy indicator of IQ, or the ‘Proxy for the Deficit Syndrome’ (PDS), a case identification method that uses a set of the PANSS or the BPRS items to determine deficit schizophrenia status.^16,17^ Finally, in the third type of proxy measures, the construct of interest is assessed indirectly through demographic and socioeconomic variables. Some examples are residential postal codes as a measure of socioeconomic status; total healthcare costs as an indirect estimate of the intensity of health service use; or the sum of being unmarried, unemployed and having lower education as an indicator of poor premorbid psychosocial adjustment.^18–20^ The latter are particularly useful in reviewing medical records or in EHR-based studies. However, despite their potential, the number of validated proxies available is still scant, and their use in mental health research is not yet widespread.

## Aims

The aim of this study was to develop and validate proxy measures of psychotic and affective symptoms based on the short versions of the PANSS, YMRS and MADRS scales, using unstructured clinical notes from EHRs. Such proxy measures would be very useful for assessing the severity of clinical features in retrospective chart reviews and in observational studies of real-world practice in patients with psychosis. They could, therefore, facilitate the implementation of large-scale studies based on representative samples of people with a psychotic disorder.

## Method

### Study population and inclusion/exclusion criteria

A multicentre, case-register study was conducted to examine the correlations between the short versions of the PANSS, YMRS and MADRS scales and their proxy versions. The sample was recruited from patients aged 18–55 years attending the mental health services (emergency, in-patient and out-patient settings) of 12 public hospitals in Andalusia (southern Spain) for treatment of first-episode psychosis (FEP). In addition, an independent cohort of patients from the Treatment and Early Intervention in Psychosis Program (TIPP-Lausanne; Switzerland) was used for external validation of the proxy instruments.^21^ The sum of all of these hospitals comprised an epidemiological catchment area of 5.5 million (approximately 3 million aged 18–55 years). Psychosis was confirmed with the Mini-International Neuropsychiatric Interview in the primary cohort and the Comprehensive Assessment of At Risk Mental States in the TIPP-Lausanne.^22,23^ All patients met the ICD-10 diagnostic criteria for psychotic disorders (F1x.4, F1x.5, F1x.7, F20–29, F30.2, F31.2, F31.5, F31.6, F32.3, F33.3 and F53.1 codes).^24^ Exclusion criteria were organic psychoses, past diagnosis of psychotic disorder, severe or unstable medical condition, history of traumatic brain injury, cognitive impairment or major neurological disease, or any level of intellectual disability or autism spectrum disorder. The Strengthening the Reporting of Observational Studies in Epidemiology (STROBE) statement guidelines were followed (see checklist in Supplementary Table 1 available at https://doi.org/10.1192/bjo.2023.623 in Supplementary Appendix 1).^25^ The authors assert that all procedures contributing to this work comply with the ethical standards of the relevant national and institutional committees on human experimentation and with the Helsinki Declaration of 1975, as revised in 2008. Patient consent was obtained in accordance with the requirements of the Andalusian Biomedical Research Ethics Committee (approval number 2184-*N*-21; informed consent was not required because of the use of de-identified data) and the Human Research Ethics Committee of the Canton Vaud (protocol number 2020-00272; verbal consent was witnessed and formally recorded). All procedures involving human participants in this research were approved by both ethics committees.

### Selected demographic, clinical and psychometric variables

Information on patient sociodemographic, clinical and psychometric characteristics was acquired at study enrolment by a clinician-administered questionnaire designed by the authors. Sociodemographic data collected included age, gender, ethnicity (European White or other), marital status (married/partnership or unmarried), living situation (alone or with others), education (higher education and secondary/lower education) and occupation (employed, unemployed or student). Clinical characteristics collected included age at onset of psychosis, duration of untreated psychosis (following the methodology outlined in the Nottingham Onset Schedule^26^), first-degree family history of psychosis, premorbid psychosocial adjustment (assessed with a validated proxy measure to discriminate patients with from those without poor premorbid adjustment^20^), psychiatric history, history of harmful or hazardous substance use (alcohol, cannabis or other illicit drugs), type of psychotic disorder (according to ICD-10 coding), presence of suicidal symptoms (including suicidal ideation, plans or attempts according to the Paykel Suicide Scale methodology^27–29^) and patient status (in-patient *v.* out-patient) at the time of enrolment. Psychometric measures were assessed at time of enrolment, using the shortened versions of the PANSS, YMRS and MADRS and their respective proxies.^30–32^ The time of the psychometric assessment (acute or stabilisation phase) and the experience of the clinician assessing the proxies (less than or over 5 years) were also recorded.

### Abbreviated versions of PANSS, YMRS and MDRS scales

The six-item version of the PANSS (PANSS-6) is a brief rating scale focusing on the severity of key schizophrenia symptoms. It consists of three items each for the positive and negative subscales (P1 delusions, P2 conceptual disorganisation, P3 hallucinations, N1 blunted affect, N4 social withdrawal, N6 lack of spontaneity and flow of conversation). PANSS-6 is psychometrically valid, reliable, sensitive to change, scalable and can be used to define remission in patients with psychosis.^30^ Symptomatic remission is defined as a PANSS-6 total score <14 and a score of mild or less (≤3) on each of its six items.^30,33^

The abbreviated version of the YMRS is used to evaluate the six core clinical symptoms of a manic episode: elevated mood (item 1), increased motor activity/energy (item 2), decreased need for sleep (item 4), increased rate or amount of speech (item 6), language/thought disorder (racing thoughts or flight of ideas) (item 7) and insight (excessive involvement in risky activities) (item 11).^31^ The YMRS-6 is a brief instrument that measures treatment response in manic and mixed episodes adequately. Clinical remission is defined as a score of <4.^31,34^

The MADRS-6 is a six-item subscale score of the original test, widely acknowledged to focus on the core symptoms of depression: apparent sadness (item 1), reported sadness (item 2), inner tension (item 3), lassitude (item 7), inability to feel (item 8) and pessimistic thoughts (item 9).^32^ The MADRS-6 is a reliable instrument for assessing antidepressant efficacy in affective disorders. A score of <5 points is considered clinical remission.^32,34^

### Proxy tools for the assessment of the PANSS-6, YMRS-6 and MADRS-6, and methodology for measuring in EHR-based studies

For scoring the PANSS-6, YMRS-6 and MADRS-6 proxies, clinicians carefully read the patient's EHR considering the setting in which the clinical note was written (emergency unit, in-patient admission, community follow-up, etc.). Patients with non-affective psychosis were assessed with the PANSS-6 proxy, and those with affective psychosis were also assessed with the YMRS-6 proxy and/or the MADRS-6 proxy, depending on whether it was a case of mania, depression or mixed episodes with psychotic symptoms. When symptoms were not reported as such in the medical record (relatively commonplace in routine practice because of time constraints during visits), clinicians had to complete the missing data by contextualising them based on the clinical setting (emergency department, in-patient admission, community follow-up, etc.), their experience and knowledge of the psychotic disorder being assessed.

**Fig. 1:**
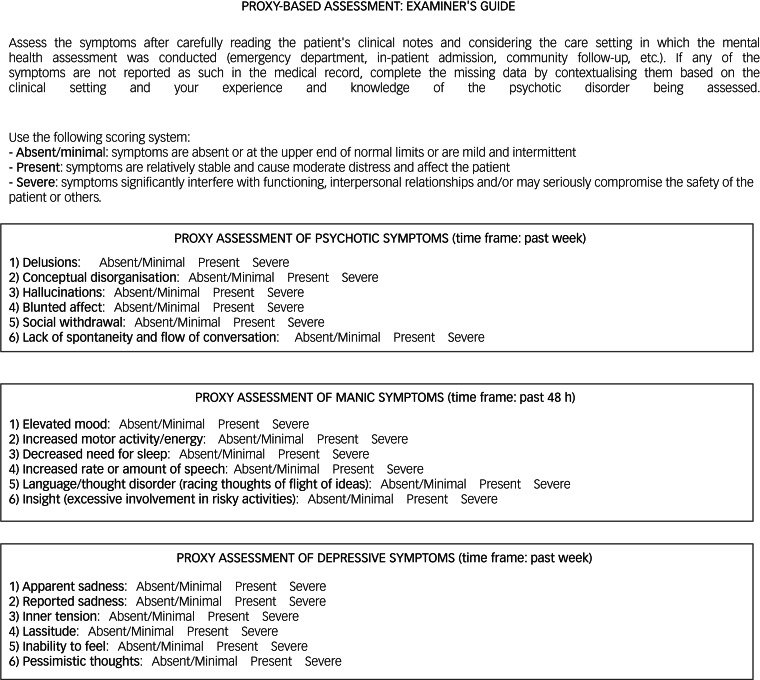
The proxies and their scoring criteria.

**Fig. 2:**
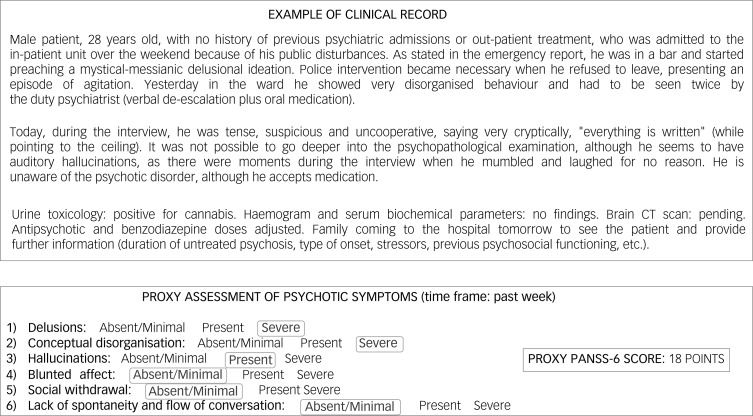
Example of proxy scoring method. CT, computed tomography; PANSS-6, Positive and Negative Syndrome Scale.

The proxies and their scoring are presented in Fig. 1. Proxies for psychotic and affective features were constructed with a symptom checklist based on items from the PANSS-6, YMRS-6 and MADRS-6. Symptoms were classified as absent/mild, present or severe according to the following criteria: (a) ‘absent/mild’ when symptoms are absent or at the upper end of normal limits or are mild and intermittent; (b) ‘present’ when symptoms are relatively stable and cause moderate distress and affect the patient; and (c) ‘severe’ when symptoms significantly interfere with functioning, interpersonal relationships and/or may seriously compromise the safety of the patient or others. The PANSS-6 proxy scores were 1 (absent/mild), 3 (present) and 6 (severe), with total scores ranging from 6 to 36 points. Clinical remission for the proxy PANSS-6 was defined as a score of 6, with all symptoms classified as absent/mild. The YMRS-6 and MADRS-6 proxies were scored as 0 (absent/mild), 3 (present) and 6 (severe), with total scores ranging from 0 to 36 points. For these proxies, a score of ≤3 was defined as clinical remission, with all but one of the symptoms classified as absent or mild. An example of the scoring method is shown in Fig. 2.

### Standardised procedure for the validation of PANSS-6, YMRS-6 and MADRS-6 proxy measures

The proxy measures were validated in the context of real-world practice with patients with FEP. Clinicians participating in the study were trained in the use of the assessment tools and followed a manual with instructions designed by the first author. Thus, during a clinical interview, a first clinician recorded a clinical note describing the patient's mental condition, and a second clinician completed the psychometric assessment with the PANSS-6, YMRS-6 and/or MADRS-6 as appropriate, depending on whether the patient had a non-affective or affective psychosis. A third clinician, not present during the clinical interview and blinded to the scale scores, then carefully read the clinical notes in the patient's EHR and examined the severity of symptoms with the proxy tools. The concordance between the scale and proxy scores, using a variety of statistical methods, determined the validity of the proxy instruments.

### DIRAYA: the electronic health information system of the Andalusian Health Service

The main cohort's EHR data were collected from DIRAYA, the digital health record software used in the Andalusian public health system (Spain). The DIRAYA is an EHR system that integrates all of the health information for each patient into a single regional record linking all of the clinical data from primary care centres, specialist out-patient clinics, hospitals, emergency services and pharmacies, including electronic drug prescriptions, telemedicine, referrals, radiology, laboratory tests, appointments, etc. The DIRAYA is one of the largest registration systems in Europe, containing clinical information on the more than 8 million people in the Andalusian public health network, and is therefore a very valuable resource for epidemiological studies.^35,36^

### Data analysis

Descriptive statistics for patient characteristics were expressed as percentages, mean or median, and s.d. or interquartile range (IQR), as appropriate. Normal distribution was tested with the Kolmogorov–Smirnov test. Differences between PANSS-6, YMRS-6 and MADRS-6 and their respective proxy versions were examined by univariate and multivariate analyses. An *a priori* power analysis was performed to calculate the appropriate sample size. With an expected correlation coefficient of 0.6, an assumed type 1 error of 0.05 and a type 2 error rate of 0.20, each of the proxies required a minimum sample size of 19 to achieve adequate statistical power. Either the Pearson's or Spearman's coefficient was used for binary correlations between scores on each instrument, depending on whether or not the normality criteria were met. Partial correlation coefficients were performed to estimate the relationships between the psychometric scales and proxy scores, controlling for potential confounders such as type of psychosis (non-affective or affective), time of assessment (acute or stable) and clinician experience in scoring the proxies (less than or over 5 years). Scatter plots were drawn to display the correlations between variables. The extent to which the proxies predicted clinical remission was determined by regression analysis (using the above cut-off points for each scale and its corresponding proxy). Odds ratios and 95% confidence intervals were estimated by logistic regression. The Nagelkerke *R*^2^ was used to assess the variance explained by the model. A classification table and area under the receiver operating characteristic curve (AUC) were used to evaluate the accuracy and discriminatory ability of the proxies. Internal validation was performed with bootstrapping (2000 resamples). External validation was performed by repeating the same statistical analyses described above in the TIPP-Lausanne cohort patients with early psychosis. Thus, the replicability of the findings in a different data-set would ensure the generalisability of the proxy tools. Interrater reliability of the proxy method was tested by intraclass correlation coefficient (ICC), two-way random effects model and absolute agreement. To this end, one of the participating patients diagnosed with a psychotic episode with mixed affective features was selected at random to assess the interrater reliability of the PANSS-6, YMRS-6 and MADRS-6 proxies, using a single case history. The ratings of this patient's clinical notes were examined by 20 different raters across all of the participating centres for ICC calculation. Missing data were managed with the listwise deletion method. Significance was set at *P* < 0.05. All statistical analyses were conducted with SPSS Statistics version 24 for MacOS (IBM Corporation, Armonk, New York, USA) and MedCalc Statistical Software version 20 for Mac OS (MedCalc Software, Ostend, Belgium; see https://www.medcalc.org).

## Results

### Patient sample characteristics

Table 1 shows the patient sociodemographic and clinical characteristics. From April 2022 to December 2022, 116 patients with FEP diagnosed following ICD-10 criteria (codes F1x.4, F1x.5, F1x.7, F20-29, F30.2, F31.2, F31.5, F31.6, F32.3, F33.3 and F53.1) were recruited. Their median age was 26 years (IQR 22–35 years) and 69.8% (*n* = 81) were men. The majority were European White (83.6%, *n* = 97) and unmarried (79.3%, *n* = 92). Of the total sample, 17.2% (*n* = 20) were living alone, 25% (*n* = 29) had a higher education and 38.8% (*n* = 45) were unemployed. The median age at onset of psychosis was 25.5 years (IQR 21–34 years) and the median duration of untreated psychosis was 1 month (IQR 1–5 months). Nineteen patients (16.4%) had a first-degree family history of psychosis. Poor premorbid adjustment was noted in 31.9% (*n* = 37) of the sample, 45.7% (*n* = 53) had past psychiatric records and 57.8% (*n* = 67) had a concurrent history of harmful or hazardous substance use. The most common diagnosis according to ICD-10 criteria was schizophrenia and schizophreniform disorder (31%, *n* = 36) (see Table 1 for further details). Suicidal symptoms were observed in 22.4% (*n* = 26) of the sample. The majority of the patients with FEP (76.7%, *n* = 89) were hospital in-patients at the time of recruitment.

**Table 1:** Sociodemographic and clinical characteristics

	Total sample (*n* = 116)
Sociodemographic characteristics	
Age (years), median (IQR)	26 (22–35)
Gender (male), *n* (%)	81 (69.8%)
Ethnicity (European White), *n* (%)	97 (83.6%)
Marital status (unmarried), *n* (%)	92 (79.3%)
Cohabitation (living alone), *n* (%)	20 (17.2%)
Education (higher education, *n* (%)	29 (25%)
Occupation (unemployed), *n* (%)	45 (38.8%)
Clinical characteristics	
Age at psychotic onset (years), median (IQR)	25.5 (21–34)
DUP (months), median (IQR)	1 (1–5)
Family history of psychosis (yes), *n* (%)	19 (16.4%)
Premorbid psychosocial adjustment (poor premorbid adjustment^a^), *n* (%)	37 (31.9%)
Past psychiatric record (yes), *n* (%)	53 (45.7%)
History of substance use, *n* (%)	67 (57.8%)
Psychotic disorder (ICD-10 codes), *n* (%)	
Schizophrenia and schizophreniform disorder (F20)	36 (31%)
Acute and transient psychotic disorder (F23)	20 (17.2%)
Mania with psychotic symptoms (F30.2)	12 (10.3%)
Substance-induced psychotic disorder (F1x.5)	12 (10.3%)
Other and unspecified psychotic disorders (F28–29)	12 (10.3%)
Depressive disorder with psychotic features (F32.3, F33.3)	9 (7.8%)
Bipolar disorder with psychotic features (F31.2, F31.5, F31.64)	6 (5.2%)
Persistent delusional disorder (F22)	4 (3.4%)
Schizotypal disorder (F21)	3 (2.6%)
Schizoaffective disorder (F25)	2 (1.7%)
Suicidal symptoms, *n* (%)	26 (22.4%)
In-patient status at the time of recruitment, *n* (%)	89 (76.7%)

IQR, interquartile range; DUP, duration of untreated psychosis.

a. The sum of the following sociodemographic variables was used as an indicator of poor premorbid adjustment: being unmarried, unemployed and low education.

### Psychometric assessment of patients

Psychometric characteristics of the sample are summarised in Table 2. A total of 116 assessments of psychotic symptoms (with the PANSS-6 and its proxy), 26 assessments of manic symptoms (with the YMRS-6 and its proxy) and 31 assessments of depressive symptoms (the MADRS-6 and its proxy) were made. The median PANSS-6 score was 15 (IQR 11–20.75) and the PANSS-6 proxy median was 14 (IQR 8–18.75). In terms of remission criteria, 30.2% (*n* = 35) of patients assessed with the PANSS-6 were classified as in symptomatic remission versus the 23.3% (*n* = 27) who met the proxy criteria. The median was 3.5 (IQR 0–20) for the YMRS-6 and 3 (IQR 0–24.75) for its proxy. Twelve patients (46.2%) met YMRS-6 remission criteria, and 14 (53.8%) were classified as in clinical remission by the proxy. The median MADRS-6 score was 9 (IQR 2–19) and the MADRS-6 proxy median score was also 9 (IQR 3–15). The MADRS-6 classified 29% (*n* = 9) of patients as in clinical remission, and 32.3% (*n* = 10) were classified as in clinical remission by the proxy. More than half of the patients (57.8%, *n* = 67) were assessed in the acute phase of psychosis and over three-fourths (76.7%, *n* = 89) had non-affective psychotic disorders. The majority of the proxies (61.2%, *n* = 71) were completed by experienced clinicians (over 5 years).

**Table 2:** Psychometric characteristics

Psychometric assessments	
PANSS-6 score (*n* = 116), median (IQR)	15 (11–20.75)
PANSS-6 remission criteria,^a^ *n* (%)	35 (30.2%)
YMRS-6 score (*n* = 26), median (IQR)	3.5 (0–20)
YMRS-6 remission criteria,^b^ *n* (%)	12 (46.2%)
MADRS-6 score (*n* = 31), median (IQR)	9 (2–19)
MADRS-6 remission criteria,^c^ *n* (%)	9 (29%)
Proxy-based assessments	
Proxy PANSS-6 score (*n* = 116), median (IQR)	14 (8–18.75)
Proxy PANSS-6 remission criteria,^a^ *n* (%)	27 (23.3%)
Proxy YMRS-6 score (*n* = 26), median (IQR)	3 (0–24.75)
Proxy YMRS-6 remission criteria,^b^ *n* (%)	14 (53.8%)
Proxy MADRS-6 score (*n* = 31), median (IQR)	9 (3–15)
Proxy MADRS-6 remission criteria,^c^ *n* (%)	10 (32.3%)

PANSS-6, Positive and Negative Syndrome Scale; IQR, interquartile range; YMRS-6, Young Mania Rating Scale; MADRS-6, Montgomery–Åsberg Depression Rating Scale.

a. Symptomatic remission was defined as a PANSS-6 score <14 (with a score of ≤3 on each of the items) and a proxy PANSS-6 score of 6.

b. Symptomatic remission was defined as a YMRS-6 score <4 and a proxy YMRS-6 score ≤3.

c. Symptomatic remission was defined as a MADRS-6 score <5 and a proxy MADRS-6 score ≤3.

### Correlation and regression analyses between scales and proxies

**Fig. 3:**
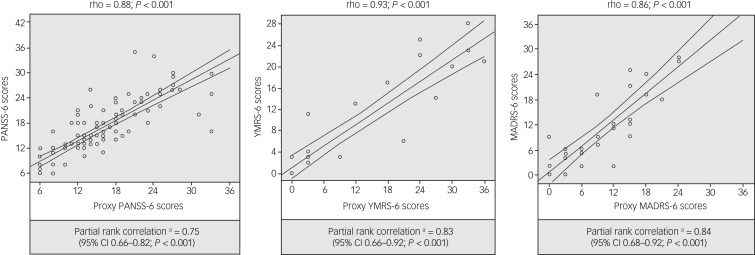
Scatter plots and correlation analyses between scale scores and their respective proxy scores. ^a^After controlling for type of psychosis (non-affective or affective), time of assessment (acute or stable) and clinician experience (<5 or 5 years). MADRS-6, Montgomery–Åsberg Depression Rating Scale; PANSS-6, Positive and Negative Syndrome Scale; YMRS-6, Young Mania Rating Scale.

Scatter plots and correlation analyses are shown in Fig. 3. The Spearman correlation coefficient (rho) between the PANSS-6 and its proxy was 0.88 (95% CI 0.83–0.91; *P* < 0.001). After controlling for type of psychosis (non-affective or affective), time of assessment (acute or stable) and clinician experience (less than or over 5 years), the partial rank correlation coefficient was 0.75 (95% CI 0.66–0.82; *P* < 0.001), indicating a strong positive linear relationship between the two measures. Regression analysis showed that the proxy PANSS-6 very strongly predicted psychotic remission (odds ratio 231.11, 95% CI 27.94–1911.66; *P* < 0.001). The Nagelkerke's *R*^2^ was 0.68, which means that the model accounted for 68% of the variance. The classification table showed that 91.4% of patients with FEP were classified correctly as in or not in remission. The AUC was 0.93 (95% CI 0.87–0.99) and the bootstrapped AUC was 0.89 (95% CI 0.83–0.95), demonstrating that the model had low overfitting and that the proxy PANSS-6 had excellent predictive power. For the YMRS-6 and its proxy, the Spearman's rho coefficient was 0.93 (95% CI 0.85–0.97; *P* < 0.001), indicating a strong positive correlation between these two variables. The partial rank correlation coefficient was 0.83 (95% CI 0.66–0.92; *P* < 0.001), showing that this strong relationship persisted after adjustment for potential confounders (type of psychosis, time of evaluation and clinician experience in scoring the proxies). Logistic regression showed that the proxy YMRS-6 predicted the psychometric criteria for remission of mania well (odds ratio 66, 95% CI 5.23–833.56; *P* = 0.001), accounting for 66% (Nagelkerke's *R*^2^) of the variance. The model showed that the proxy version correctly classified 88.5% of patients as in or not in remission according to YMRS-6 criteria. The AUC was 0.89 (95% CI 0.74–1) and the bias-corrected AUC after bootstrapping was 0.88 (95% CI 0.70–1), indicating that the model had minimal overfitting and that the YMRS-6 proxy had excellent discriminatory performance. For depressive symptoms, the Spearman coefficient for the correlation between the MADRS-6 and the proxy MADRS-6 was 0.86 (95% CI 0.72–0.93; *P* < 0.001). This strong positive linear relationship remained significant after adjustment for the covariates listed above, with a partial rank coefficient of 0.84 (95% CI 0.68–0.92; *P* < 0.001). Regression analysis confirmed this strong relationship, as the proxy MADRS-6 predicted the clinical remission of depression well (odds ratio 22.17, 95% CI 3.04–161.84; *P* = 0.002). Nagelkerke's *R*^2^ was 0.46. The classification table showed that the MADRS-6 proxy correctly distinguished 83.9% of patients in and not in remission according to the MADRS-6 remission criteria. The AUC was 0.80 (95% CI 0.62–0.99) and the bootstrapped optimism-corrected AUC was 0.76 (95% CI 0.52–1). This indicated low overfitting of the model and good predictive power of the proxy version of the MADRS-6.

### External validation and interrater reliability of the proxy tools

A total of 30 assessments of positive, manic and depressive symptoms were made of patients from the TIPP-Lausanne cohort to provide external validation of the above findings. More than half of the proxies were assessed during the stabilisation phase and in cases of non-affective psychotic disorders (70%, *n* = 21). The majority were completed by clinicians with less than 5 years of experience (66.6%, *n* = 20). The sociodemographic, clinical and psychometric characteristics of these patients are summarised in Supplementary Table 2, whereas scatter plots and correlation analyses are shown in Supplementary Fig. 1 in Supplementary Appendix 1. The Spearman correlation coefficient between the PANSS-6 and the proxy was 0.82 (95% CI 0.65–0.91; *P* < 0.001), whereas the partial rank correlation coefficient after adjustment for potential confounders (type of psychosis, time of assessment and clinician experience in scoring the proxies) was 0.66 (95% CI 0.39–0.82; *P* < 0.001). Regression analysis showed that the proxy PANSS-6 significantly predicted the psychometric criteria for PANSS-6 remission (odds ratio 20, 95% CI 2.04–196.37; *P* = 0.010). The Nagelkerke's *R*^2^ was 0.40, the model correctly classified 76.7% of patients in and not in remission, and the AUC was 0.75 (95% CI 0.57–0.94). The Spearman coefficient for the correlation between the YMRS-6 and its proxy was 0.76 (95% CI 0.54–0.88; *P* < 0.001), and this strong positive relationship remained significant after adjusting for the above-mentioned covariates, with a partial rank coefficient of 0.77 (95% CI 0.57–0.88; *P* < 0.001). Regression analysis showed that the proxy YMRS-6 significantly predicted the psychometric criteria for remission from mania (odds ratio 26, 95% CI 3.69–183.42; *P* = 0.001), with a Nagelkerke's *R*^2^ for the model of 0.52. It correctly classified 83.3% of patients in and out of remission, and the AUC was 0.84 (95% CI 0.68–0.99). The Spearman coefficient for the correlation between the MADRS-6 and its proxy was 0.83 (95% CI 0.66–0.91; *P* < 0.001), whereas the partial rank correlation coefficient, after controlling for potential confounders, was 0.84 (95% CI 0.69–0.92; *P* < 0.001). Regression analysis showed that the proxy version significantly predicted MADRS-6 remission criteria (odds ratio 28, 95% CI 2.82–277.96; *P* = 0.004). Nagelkerke's *R*^2^ was 0.47, the model correctly discriminated 80% of patients in and not in remission according to MADRS-6 criteria, and its AUC was 0.82 (95% CI 0.67–0.98). Finally, the overall ICC of the proxies calculated to quantify agreement between raters was 0.81 (95% CI 0.52–0.99; *P* < 0.001), indicating that the proxy method had good interrater reliability.

## Discussion

The aim of this study was to develop and examine the validity and reliability of PANSS-6, YMRS-6 and MADRS-6 proxy measures based on information in clinical records from EHRs in a real-world setting. The main findings were that the proxy versions were strongly correlated with their respective standardised scales and had good accuracy and discriminatory power in distinguishing between patients in and not in remission, with high interrater reliability. These findings were not altered when the effects of other explanatory variables, such as type of psychosis (non-affective or affective), time of assessment (acute or stabilisation phase) and clinician experience (less than or over 5 years), were controlled for. The proxies were also tested in an independent FEP cohort, showing good external validation, which suggests that the proxy method for assessing psychotic, manic and depressive symptoms could be generalised to any other EHR data-set. To the best of our knowledge, this is the first study validating EHR-based proxy tools for the psychometric assessment of psychotic and affective symptoms in patients with FEP under routine clinical practice conditions.

As there are no other validated clinician-administered proxy reporting instruments for the assessment of psychotic and affective symptoms in patients with FEP, the results of the development and validation of our proxy tools had to be compared with other available validated, clinician-rated proxy measures in the field of psychosis. Thus, the correlation coefficients for the proxy PANSS-6, YMRS-6 and MADRS-6, which ranged from 0.75 to 0.93, were considerably higher than the 0.57 found in validating a proxy measure of the Premorbid Adjustment Scale (PAS).^20^ Accuracy of remission status classification by the PANSS-6, YMRS-6 and MADRS-6 proxies (83.9–91.4%) was similar to the 82% achieved with the PAS proxy and the 87% achieved with the proxy case identification tool for deficit schizophrenia (PDS).^17,20^ Finally, AUCs indicating the discriminative power of the PANSS-6, YMRS-6 and MADRS-6 proxies ranged from 0.80 to 0.93, which was slightly better than the 0.78 achieved by the PAS proxy and the 0.73 achieved by a proxy measure of PANSS remission criteria based on a CGI-Improvement Scale value of 1.^20,37^ The interrater reliability of our proxy methodology (ICC = 0.81) was similar to that found by Fenton and McGlashan,^38^ when they retrospectively scored the Schedule for the Assessment of Positive Symptoms (SAPS) and the Schedule for the Assessment of Negative Symptoms (SANS) by reviewing the admission records of patients with FEP. Although those authors did not validate their methodology, they found high interrater agreement of the SAPS (ICC = 0.85) and SANS (ICC = 0.83) proxies from manual chart review.^38^ All of the above comparisons suggest that our proxy versions of the PANSS-6, YMRS-6 and MADRS-6 meet validity and reliability standards, and can be used to measure the severity of psychotic and affective symptoms in EHRs.

### Methodological limitations

Our findings should be viewed in the light of the following three main limitations. First, there were sample size-related limitations, particularly in the validation of the YMRS and MADRS proxies, which yielded some very high correlations in the univariate analysis that exceeded the reliability of the scales. This phenomenon can occur by chance and the real value, although probably still very high, is likely to be lower.^39^ Notwithstanding, our sample size was still larger than the minimum required to achieve statistical power for correlation analyses. Second, there were limitations arising from the simplicity of the proxies, in that although they assess the severity of the core features of psychotic, manic and depressive symptoms, they do not capture other psychopathological features with significant predictive value in patients with psychosis, such as psychomotor disturbances or first-rank symptoms of schizophrenia.^40^ Third, despite the simplicity of these proxy instruments, another limitation of the methodology would be the time required to accurately assess symptom severity and estimate missing data by clinical contextualisation through careful reading of patients’ clinical notes. Fourth, the quality of the clinical notes in the primary cohort may have been somewhat biased because clinicians who wrote them knew that their notes would later be used to construct a proxy measure, and this could have influenced how they recorded their findings on patients’ mental states. In this regard, we would like to emphasise that the EHR clinical notes on which the proxy ratings were based were similar to those routinely collected in real-world practice, and that this potential bias was not present in the external validation, as the notes on the TIPP-Lausanne cohort were written before it joined this study. Finally, there is the inherent limitation of a cross-sectional design in capturing changes in patients’ psychopathology over time. However, it is worth noting that our proxy methodology discriminates well between patients in and not in remission, and that the time of psychopathological assessment (acute or stabilisation phase) was a covariate in the multivariate analyses.

### Clinical implications and future directions

Despite these limitations, our proxies for the abbreviated PANSS, YMRS and MADRS have meaningful implications. They are instruments based on real-life settings valuable for large-scale collaborative research, as they could make different databases psychometrically compatible. They could also be useful tools for homogenising clinical information in case report-based systematic reviews. Beyond the research framework, they would also be useful for monitoring the progress of patients with severe mental disorders in clinical practice, and for examining the effectiveness of mental health treatment programmes. Future directions in this area should study whether these proxy measures can be administered by other healthcare professionals (such as general practitioners, nurses or social workers), or whether they would be valid for use with children and adolescents. Similarly, as proxies were validated in high-income countries with well-structured, resourced mental health services, they should also be validated in low- and middle-income countries with fewer mental health resources, where not everyone with FEP has access to early intervention programmes. Finally, it is important to highlight the opportunities that artificial intelligence and natural language processing would offer in analysing the unstructured free text in the EHRs to automatically provide proxy estimates of patients’ clinical status. In this respect, it would be of great interest to integrate our proxy measures with SAVANA's EHRead and CRIS-CODE technology.^41,42^ Such integration with artificial intelligence with natural language processing algorithms could somewhat alleviate the ongoing ethical dilemma in biomedical EHR-based research regarding the extraction of data from patients’ medical records without informed consent.^43,44^

In summary, we conclude that standardised psychometric assessments are essential for patients with FEP. Therefore, our proposal of a proxy instrument based on clinical records for the assessment of psychotic and affective symptoms offers a feasible, valid and reliable alternative to standard psychometric procedures in real-world practice settings. This would greatly expand opportunities for observational studies using routinely collected health data, and would be particularly useful in research-based on EHRs.

## Supporting information

López-Díaz et al. supplementary material López-Díaz et al. supplementary material

## Data Availability

The data that support the findings of this study are available from the corresponding author, Á.L.-D., upon reasonable request.
